# DNA G-quadruplexes for native mass spectrometry in potassium: a database of validated structures in electrospray-compatible conditions

**DOI:** 10.1093/nar/gkab039

**Published:** 2021-02-08

**Authors:** Anirban Ghosh, Eric Largy, Valérie Gabelica

**Affiliations:** Univ. Bordeaux, CNRS, INSERM, ARNA, UMR 5320, U1212, IECB, F-33000 Bordeaux, France; Univ. Bordeaux, CNRS, INSERM, ARNA, UMR 5320, U1212, IECB, F-33000 Bordeaux, France; Univ. Bordeaux, CNRS, INSERM, ARNA, UMR 5320, U1212, IECB, F-33000 Bordeaux, France

## Abstract

G-quadruplex DNA structures have become attractive drug targets, and native mass spectrometry can provide detailed characterization of drug binding stoichiometry and affinity, potentially at high throughput. However, the G-quadruplex DNA polymorphism poses problems for interpreting ligand screening assays. In order to establish standardized MS-based screening assays, we studied 28 sequences with documented NMR structures in (usually ∼100 mM) potassium, and report here their circular dichroism (CD), melting temperature (*T*_m_), NMR spectra and electrospray mass spectra in 1 mM KCl/100 mM trimethylammonium acetate. Based on these results, we make a short-list of sequences that adopt the same structure in the MS assay as reported by NMR, and provide recommendations on using them for MS-based assays. We also built an R-based open-source application to build and consult a database, wherein further sequences can be incorporated in the future. The application handles automatically most of the data processing, and allows generating custom figures and reports. The database is included in the *g4dbr* package (https://github.com/EricLarG4/g4dbr) and can be explored online (https://ericlarg4.github.io/G4_database.html).

## INTRODUCTION

Nucleic acids constitute the fundamental biomolecular machinery to transfer genetic information, but are also involved in the regulation of gene expression (1). Besides the canonical double helix, nucleic acids can adopt various non-canonical structures, i.e. triplexes, slipped hairpins, four-way junctions, left-handed Z-form, cruciform, G-quadruplexes or i-motifs (2). G-quadruplexes (G4s) have been the subject of intense structural and biological research, given their roles in gene regulation and other related cellular processes (3,4). G4s indeed have important biological effects in replication, transcription, translation, mutagenesis, genome damage repair, telomere maintenance, or RNA splicing (3,5,6). Their key role in different cellular processes makes them crucial drug targets for diseases (6–8). Besides biological roles, G4s have also numerous other applications in theranostics, supramolecular chemistry, or nanotechnology (6,9–12).

The building block is a G-quartet wherein four guanines adopt a square planar arrangement stabilized by eight Hoogsteen hydrogen bonds (4). The stacking of adjacent G-quartets is further stabilized by coordination with monovalent or divalent cations positioned in-between G-quartets (4,13). The guanine repeats are connected by loops. The G4 topologies are conveniently categorized as parallel (four strands in the same direction, all-anti homo-stacking), antiparallel (two strands in one direction and two in the other direction, alternate syn/anti hetero-stacking) and hybrid (three strands in one direction and one strand in the reverse direction, combination of homo and hetero stacking). Further sub-classes can be defined depending on the number of G-quartets, or which loop is lateral, diagonal or propeller. These topologies are themselves linked with the conformation of the glycosidic bond angle between the guanine base and sugars, to the stacking arrangement giving rise to specific circular dichroism signals, and to the groove width distribution (14,15).

Various analytical methods are routinely used to characterize G4 structures and their interaction with ligands (small molecule, proteins, cations, co-solvents etc.). Each experimental technique has inherent limitations in terms of analytes (e.g. oligonucleotide size, concentration, thermodynamic stability, labeling), buffers (e.g. cation nature and concentration, ionic strength, pH, volatility), and conditions (e.g. temperature, pressure). Studies on human telomeric sequences (TTAGGG repeats), in particular, have revealed that minor changes in the oligonucleotide sequence or in the buffer conditions can alter the structure. At least eight different types of intramolecular G4 topologies were identified to date and some sequences are inherently polymorphic (13,16,17). In the absence of external factors (i.e. co-solvents, proteins), the nature and concentration of cations predominantly affect the G4 conformation of a given sequence (18,19). A seminal example of this issue is the 22-mer human telomeric sequence (22AG in this manuscript), which has been assigned as a parallel G4 by crystallography in potassium conditions (PDB: 1KF1), an antiparallel G4 by NMR in sodium conditions (PDB: 143D), and a mixture of topologies (hybrid & antiparallel) in circular dichroism of potassium-containing solutions (20–22). The comparison of results obtained by different groups, using different methods and/or experimental conditions might therefore not always be directly possible, and should always be questioned.

In this manuscript, we attempt to facilitate such comparisons by native electrospray mass spectrometry (ESI-MS) (23–25). Native MS of nucleic acids and proteins is often performed in ammonium acetate (NH_4_OAc), but although NH_4_OAc is compatible with G4 formation (24,26), potassium is more physiologically relevant, and consequently more G4 structures have been solved in K^+^ solution. To directly compare native MS data to the literature, and to work with the physiologically relevant cation, it is therefore desirable to perform ESI-MS of potassium-containing samples. Since 2014, we use ESI-MS solutions containing up to 1 mM KCl while the ionic strength is ensured by (typically 100 mM) trimethylammonium acetate (TMAA), which can also more efficiently suppress nonspecific alkali adducts than NH_4_OAc (23). The number of K^+^ ions bound to the sequence (*n*) is related to the number of G-quartet in the observed sub-ensemble (*n* + 1), and thus the MS-derived K^+^ binding constants are linked to the G-quadruplex folding constants (27). This was exploited for equilibrium, kinetics, thermal denaturation and ligand binding studies (22,28,29).

These electrospray-compatible solution conditions have not yet been used for systematic studies such as ligand screening. One possible limitation is that in 1 mM K^+^ all the quadruplexes are less stable than in high salt (∼100 mM K^+^) and as a result misfolded/alternative folded & non folded species can form to a significant extent. Alternative sample preparation methods including co-solvents (hexafluoroisopropanol, isopropanol) have been proposed to increase the signal-to-noise ratio, but the risk is to induce conformational changes (25,30,31). Therefore, in order to interpret the MS results in the light of an NMR- derived structure in K^+^ we need to systematically verify by solution spectroscopy (CD, UV melting, and ^1^H NMR) that the conformation reported in high salt conditions is also the one present in native MS conditions. The objective of the present study is to build a database of G4 sequences with sufficient stability and validated folds (based on UV melting, circular dichroism and NMR spectroscopies) in 1 mM KCl + 100 mM TMAA assay conditions. This will help short-listing sequences for future MS-based ligand screening studies in terms of structural selectivity.

## MATERIALS AND METHODS

### Materials

Oligonucleotides were purchased in lyophilized form with RP cartridge purification from Eurogentec (Seraing, Belgium). They were dissolved in nuclease-free grade water from Ambion (Ambion, Life Technologies SAS, Saint-Aubin, Franc) to have a target concentration of ∼500 μM. The concentration of the stock solutions was determined using absorbance at 260 nm and molar extinction coefficients calculated using the nearest-neighbor model in its traditional format (Equation 1), where ${\varepsilon _i}$ is the molar extinction coefficient (in M^−1^ cm^−1^) of the nucleotide in position $i$ (in the 5′ to 3′ direction), ${\varepsilon _{i,i + 1}}$ is the extinction coefficients for doublets of nucleotides in positions $i$ and $i + 1$, and ${N_b}$ is the number of nucleotides in the oligonucleotide (32,33).

(1) $$\begin{equation*}{\varepsilon _{260nm}} = \mathop \sum \limits_{i = 1}^{{N_b} - 1} {\varepsilon _{i,i + 1}}\ - \mathop \sum \limits_{i\ = \ 2}^{{N_b} - 1} {\varepsilon _i}\end{equation*}$$

The implementation of Equation (1) in the g4dbr application is provided in the g4dbr manual (Supporting information). All molar extinction coefficient values are provided in supporting information.

All sequences used in this study are listed in Table 1. Trimethylammonium acetate (TMAA, Ultra for UPLC, Fluka), potassium chloride solution (1 M concentration) (KCl, >99.999%), KH_2_PO_4_ and K_2_HPO_4_ (molecular biology grade), and D_2_O (99.9% D atom) were purchased from Sigma-Aldrich (Saint-Quentin Fallavier, France). The stock oligonucleotide solutions were diluted to 100 μM in 100 mM TMAA supplemented with 1 mM KCl in water (pH 7.0) and NMR buffer (Supplementary Table S1). They were kept at least 72 h at 4°C to ensure G-quadruplex formation. For telomeric sequences, no annealing was done, but previous studies showed that the end point will be reached after 72 h (22). For non-telomeric sequences, the solutions were annealed at 85°C for 3–4 min in a water bath then let at room temperature for 24 h before use. For UV melting and CD two buffers were used: (i) 100 mM TMAA supplemented with 1 mM KCl (MS-compatible buffer) and (ii) 5–25 mM K_2_HPO_4_/KH_2_PO_4_ buffer supplemented with KCl (pH 7.0–7.1) (Supplementary Table S1).

**Table 1.** Oligonucleotides used in this work and described in detail in the database. The respective sequence, origin, structural features in both conditions and rating are summarized below.

			Structure in ∼100 mM K^+^^a^								ESI MS condition (100 mM TMAA + 1 mM KCl)					
#nt	Sequence	Origin (gene)	PDB	Ref.	Strand orientation	Quartets	Stacking	Loops	Groove width	*T*_m_ /°C	*T*_m_ /°C	#K^+^ (MS)/%^b^	CD (1 mM vs. 100 mM K^+^)	NMR (1 mM vs. 100 mM K^+^)	Conclusion about topology in MS conditions	Rating
***Human telomeric and variants***																
24	TTGGGTTAGGGTTAGGGTTAGGGA	Human telomere	2GKU	(50)	Hybrid-1	3	II	pll	mwnm	67	41	2 (100)	Idem	Idem + minor peaks	2K^+^: Same folding as PDB	**
23	TAGGGTTAGGGTTAGGGTTAGGG	Human telomere	2JSM	(51)	Hybrid-1	3	II	pll	mwnm	64	37	0 (6), 1 (18.7), 2 (75.3)	Idem	Idem + minor peaks	2K^+^: Same folding as PDB	**
26	TTAGGGTTAGGGTTAGGGTTAGGGTT	human telomere	2JPZ	(52)	Hybrid-2	3	II	llp	mwnm	58	28	0 (16.8), 1 (18.5), 2 (64.7)	Lower intensity, same shape	Idem + minor peaks	2K^+^: Same folding as PDB, but incomplete folding	*
26	AAAGGGTTAGGGTTAGGGTTAGGGAA	Human telomere	2HY9	(53)	Hybrid-1	3	II	pll	mwnm	59	29	0 (17.8), 1 (19.8), 2 (62.4)	Idem	Idem + minor peaks	2K^+^: Same folding as PDB, but incomplete folding	*
21	GGGTTAGGGTTAGGGTTTGGG	Artificial variant	5YEY	(54)	Antiparallel	3	II	lll	nwnw	71	43	1 (16.4), 2 (83.6)	Idem	Idem	2K^+^: Same folding as PDB	**
22	GGGTTAGGGTTAGGGTTAGGGT	Human telomere	2KF8	(55)	Antiparallel	2	III	ldl	mnmw	71	41	1 (48.8), 2 (51.2)	Less homostacking (22)	Matching satisfactory + minor peaks	2K^+^: Same folding as PDB	**
22	AGGGCTAGGGCTAGGGCTAGGG	Artificial variant	2KM3	(56)	Antiparallel	2	III	lll	nwnw	67	38	0 (17.2), 1 (54.0), 2 (28.8)	Lower intensity	Matching satisfactory + minor peaks	1K^+^: Same folding as PDB, incomplete folding	*
22	TAGGGTTAGGGTTAGGGTTAGG	Human telomere	5LQG	(44)	Antiparallel	2	III	ldl	mnmw	53	36	0 (14), 1 (60), 2 (26)	Lower intensity,same shape	Partially matching + minor peaks	1K^+^: Maybe same folding as PDB, but incomplete folding	*
21	GGGTTAGGGTTAGGGTTAGGG	Human telomere	21G^c^	(57)	Mixture					70	44	1 (52.7), 2 (47.3)	Less homostacking	Polymorphic	Polymorphic but resembles 2KF8	×
22	AGGGTTAGGGTTAGGGTTAGGG	Human telomere	22AG^c^	(21,57)	Mixture					67	41	1 (49), 2 (51)	Less homostacking	Polymorphic	Polymorphic but resembles 2KF8	×
***Parallel G-quadruplexes***																
18	TTGGGTGGGTGGGTGGGT	Artificial construct	2LK7	(58)	Parallel	3	I	ppp	mmmm	> 75	71	2 (100)	Idem	Idem	2K^+^: Same folding as PDB	**
19	TAGGGCGGGAGGGAGGGAA	N-myc	2LEE	(59)	Parallel	3	I	ppp	mmmm	> 75	56	2 (100)	Idem	Idem	2K^+^: Same folding as PDB	**
19	TTGTGGTGGGTGGGTGGGT	Artificial construct	2M4P	(60)	Parallel	3	I	ppp + bulge	mmmm	> 75	52	2 (100)	Idem	Idem	2K^+^: Same folding as PDB	**
22	TAGGGTGGGTTGGGTGGGGAAT	Neisseria gonorrhoeae pilE	2LXQ	(61)	Parallel	3	I	ppp	mmmm	> 75	60	2 (100)	Idem	Idem	2K^+^: Same folding as PDB	**
22	CGGGGCGGGCCTTGGGCGGGGT	VEGF	2M27	(62)	Parallel	3	I	ppp	mmmm	> 75	45	1 (9.5), 2 (90.5)	Idem	Idem	2K^+^: Same folding as PDB	**
22	TGAGGGTGGGTAGGGTGGGTAA	c-MYC	1XAV	(63)	Parallel	3	I	ppp	mmmm	> 75	54	2 (100)	Idem	Idem	2K^+^: Same folding as PDB	**
24	TGAGGGTGGTGAGGGTGGGGAAGG	Myc2345	2A5P, 2MGN^d^	(64,65)	Parallel	3	I	ppp	mmmm	70	53	2 (100)	Idem	Idem	2K^+^: Same folding as PDB	**
26	AAGGGTGGGTGTAAGTGTGGGTGGGT	CEB25 Minisatellite	2LPW	(66)	Parallel	3	I	ppp	mmmm	> 75	53	2 (100)	Idem	Idem	2K^+^: Same folding as PDB	**
19	TAGGGAGGGTAGGGAGGGT	Myc1234	2LBY	(67)	Parallel	3	I	ppp	mmmm	> 75	44	0 (41.3), 1 (18.2), 2 (40.5)	Lower intensity	Idem+ minor peaks	2K^+^: Same folding as PDB, but incomplete folding	*
22	AGGGAGGGCGCTGGGAGGAGGG	c-kit	2O3M	(68)	Parallel	3	I	pppp	mmmm	65	33	0 (20.6), 1 (11.7), 2 (67.7)	Idem	Idem+ minor peaks	2K^+^: Same folding as PDB, but incomplete folding	*
20	TAGGGACGGGCGGGCAGGGT	Artificial construct	5NYS	(69)	Parallel	3	I	ppp	mmmm	> 75	40	0 (30.9), 1 (21.4), 2 (47.7)	Less intensity, more heterostacking	Idem	2K^+^: Same folding as PDB, but incomplete folding according to MS and CD	*
21	CGGGCGGGCGCTAGGGAGGGT	c-kit2	2KYP	(70)	Parallel	3	I	ppp	mmmm	60	42	0 ((58.1), 1 (27.8), 2 (14.1)	Less homo stacking	Not matching	Incomplete and different folding	×
22	CTGGGCGGGACTGGGGAGTGGT	HIV-1 LTR	2N4Y	(71)	Parallel	3	I	ppp+bulge	mmmm	45	<25	0 (50.9), 1 (32.4), 2 (16.7)	Less homo stacking	Not matching	Incomplete and different folding	×
22	AGGGCGGTGTGGGAATAGGGAA	KRAS	5I2V	(72)	Parallel	3	I	ppp	mmmm	48	<25	0 (54.5), 1 (25.6), 2 (19.9)	Less homo stacking	Matching satisfactory + minor peaks	May be Same folding as PDB, but incomplete folding	×
***Other G-quadruplex structures***																
19	GGGTGGGGAAGGGGTGGGT	human chl1	2KPR	(73)	Hybrid	3	II	llpp	ambiguous	> 75	53	1 (14.4), 2 (85.6)	More heterostacking	Idem	2K^+^: Same main structure as PDB, but CD differs	*
22	GGGATGGGACACAGGGGACGGG	Artificial construct	2LOD	(74)	Hybrid	3	II	pdl	mwnm	64	39	0 (17.5), 1 (26.1), 2 (56.4)	Less heterostacking	Idem + minor peaks	2K^+^: Maybe same structure as PDB, but incomplete folding and CD differs	*
19	TGGCCTGGGCGGGACTGGG	HIV-1 LTR	HIV- PRO1^e^	(75)	Antiparallel	2	III	lll	nwnw	59	29	0 (59.5), 1 (40.5)	Less stacking	Not matching	Incomplete and different folding	×
20	GGGTAGGGAGCGGGAGAGGG	RANKL	6GZN	(76)	Antiparallel	2	III	ldl	mnmw		<25	0 (35.8), 1 (33.6), 2 (30.6)	Less intensity, less homostacking	Not matching	Incomplete and different folding	×

^a^Detailed buffer compositions are given in Table S1 (Supporting information).

^b^For each system relative abundance (%) of each species under the 5– charge states have been shown in bracket

^c^In KCl solution, theses sequences are polymorphic. The name is therefore not a PDB code, but follows an internal naming convention. The provided bibliographic references showcase known conformers formed by these sequences (sometimes in presence of a ligand or sodium cations).

^d^2A5P: PDB id of the mutated (Inosine at 10^th^ position) sequence. 2MGN: PDB id of the wild type sequence in complex with a PhenDC3 ligand.

^e^There is no deposited structure for this sequence. The name is therefore not a PDB code, but follows an internal naming convention.

### Circular dichroism (CD)

All circular dichroism experiments were performed on a Jasco J‐815 spectrophotometer equipped with a JASCO CDF 426S Peltier temperature controller using a quartz cuvette (2 mm path length) at 25°C. The DNA concentration was 10 μM for all the measurements. The scanning range was 220–320 nm with 0.2 nm data pitch, 2 nm bandwidth, and 0.5-sec response. For each sample, 3 accumulations were acquired with a scan speed of 50 nm/min, then blank-corrected with the data from the corresponding buffer without DNA. The subtracted spectra were normalized to molar ellipticity coefficient (Δ*ϵ*) according to the following Equation (2):

(2) $$\begin{equation*}\Delta \varepsilon \ = \frac{\theta }{{32980\ \times C \times l}}\ \end{equation*}$$

where *θ* = CD signal in millidegrees, *C* = DNA concentration in mol/L, and *l* = path length in cm.

### Melting monitored by UV absorbance (UV-melting)

Melting temperatures were determined by measuring the changes in absorbance at 295 nm as a function of temperature, using a UVmc2 double-beam spectrophotometer (SAFAS, Monte Carlo, Monaco) equipped with a high-performance Peltier temperature controller and a thermostatable 10-cell holder, with 400-μl, 1-cm pathlength quartz cuvettes (115B-QS, Hellma GmbH & Co. KG, Müllheim, Germany). The samples contained the oligonucleotide (10 μM) in potassium phosphate or TMAA buffers, supplemented or not by potassium chloride, and were cooled to 4°C. The absorbance was monitored at 260, 295 and 335 nm on a cycle composed of a heating to 90°C at a rate of 0.2°C min^−1^, then cooling to 4°C at the same rate.

The raw absorbance data was buffer subtracted, and converted to molar extinction coefficient $\varepsilon$ (in M^-1^cm^−1^) using $\varepsilon \ = \ A/lC$, where $l$ is a path length (in cm) and $C$ the oligonucleotide concentration (in M). The melting temperatures (${T_m}$), determination for a 2-state equilibrium, and the conversion of the temperature-dependent absorbances ${A_T}$ into folded fractions ${\theta _T}$, were carried out based on a non-linear fitting-based implementation of the baseline method, using Equation (3) where $a$ and $b$ are the slopes and intercepts, respectively, of the folded ($F$) and unfolded ($U$) baselines, $R$ is the gas constant (in J K^−1^ mol^−1^), and $T$ is the temperature (in K) (34).

(3) $$\begin{eqnarray*} {A_T} &=& {\rm{ }}\left( {{a^F}T + {b^F}} \right){\rm{ }} \nonumber \\ && \times \frac{1}{{1 + exp\left( { - \frac{{\Delta {H^0}\left( {1 - \frac{T}{{{T_m}}}} \right)}}{{RT}}} \right)}} + \left( {{a^U}T + {b^U}} \right){\rm{ }} \nonumber \\ && \times \frac{{exp\left( { - \frac{{\Delta {H^0}\left( {1 - \frac{T}{{{T_m}}}} \right)}}{{RT}}} \right)}}{{1 + exp\left( { - \frac{{\Delta {H^0}\left( {1 - \frac{T}{{{T_m}}}} \right)}}{{RT}}} \right)}} \end{eqnarray*}$$

${\theta _T}$ gives a direct access to the extent of folding of an oligonucleotide (1: all molecules entirely folded, 0: all molecules entirely unfolded), allows to visually assess the ${T_m}$ ( ${\theta _t} = \ 0.5$), and normalize the data of different samples (and therefore different absorbances) to a common y-scale (34). For the non-linear fitting and the folded fraction calculation to be carried out, the data must contain both *lower* and *higher* baselines. When this was not the case (the oligonucleotide is too stable or unstable), the melting curves were simply normalized to [0;1], and no thermodynamic quantities were determined.

The derivation of Equation (3) and its implementation in the g4dbr application are provided in the g4dbr manual (Supporting information).

### Nuclear magnetic resonance (NMR)

All ^1^H-NMR experiments were carried out on a Bruker 700 MHz spectrometer (Bruker biospin) equipped with 5 mm TXI probe at 25°C. The jump-and-return water suppression is used in all experiments (35). The sweep widths were 20 ppm with a 3-sec relaxation delay with a size of 32K data points per 1D spectra. The number of scans and dummy scans was 128 and 16 respectively. The 1D raw data were processed and analyzed with Topspin 4.06 software inbuilt with the instrument. All the quadruplex sequences were 100 μM strand concentration in 100 mM TMAA + 1 mM KCl in a 5 mm NMR tube (Wilmad from CortecNet, France).

### Electrospray mass spectrometry (ESI-MS)

All ESI-MS experiments were performed in negative ion mode on an Agilent 6560 IMS-Q-TOF (Agilent Technologies, Santa Clara, CA, USA) with a dual ESI source and soft tuning conditions (36). The experiments were performed in ion mobility mode (*p*_He_ = 3.89 ± 0.01 torr, *T* = 296 ± 1 K). The source gas temperature was 200°C with fragmentor voltage at 350 V (soft conditions, by default). The injected DNA concentrations were 10 μM G-quadruplex in 100 mM TMAA and 1 mM KCl (180 μL/h flow rate with a syringe pump). The mass spectra and the arrival time distributions for the 5-ions recorded at a drift voltage of 390 V (drift tube entrance: 600 V; drift tube exit: 210 V) are described herein. A separate paper will describe the full dataset (all charge states, various conditions, conversion to collision cross section distributions and comparison with 3D models).

### Data processing, app and database

Circular dichroism, UV-melting, NMR, and native ESI-MS data filtering, normalization, fitting, and labeling was performed in g4db, an in-house Shiny application included in the g4dbr package, written in RStudio 1.3.1056 (http://www.rstudio.com), running R 4.1 (https://www.R-project.org). The database is included in the *g4dbr* package (https://github.com/EricLarG4/g4dbr) and can be explored online (https://ericlarg4.github.io/G4_database.html; http://doi.org/10.5281/zenodo.4200176). The application documentation is provided in supporting information.

## RESULTS

### Database composition and building

We examined 28 sequences (among which 10 human telomeric variants) for which a specific intramolecular G4 structures had been solved by NMR in high KCl concentration (usually ∼100 mM), with the exception of the two polymorphic sequences 22AG and 21G. The sequences will be referred to by their PDB code as listed in Table 1 (see also Supplementary Tables S2–S29). The CD, ^1^H NMR, ESI-MS spectra and UV thermal denaturation profiles are all shown in the supporting information (Supplementary Figures S1–S168).

**Figure 1.**
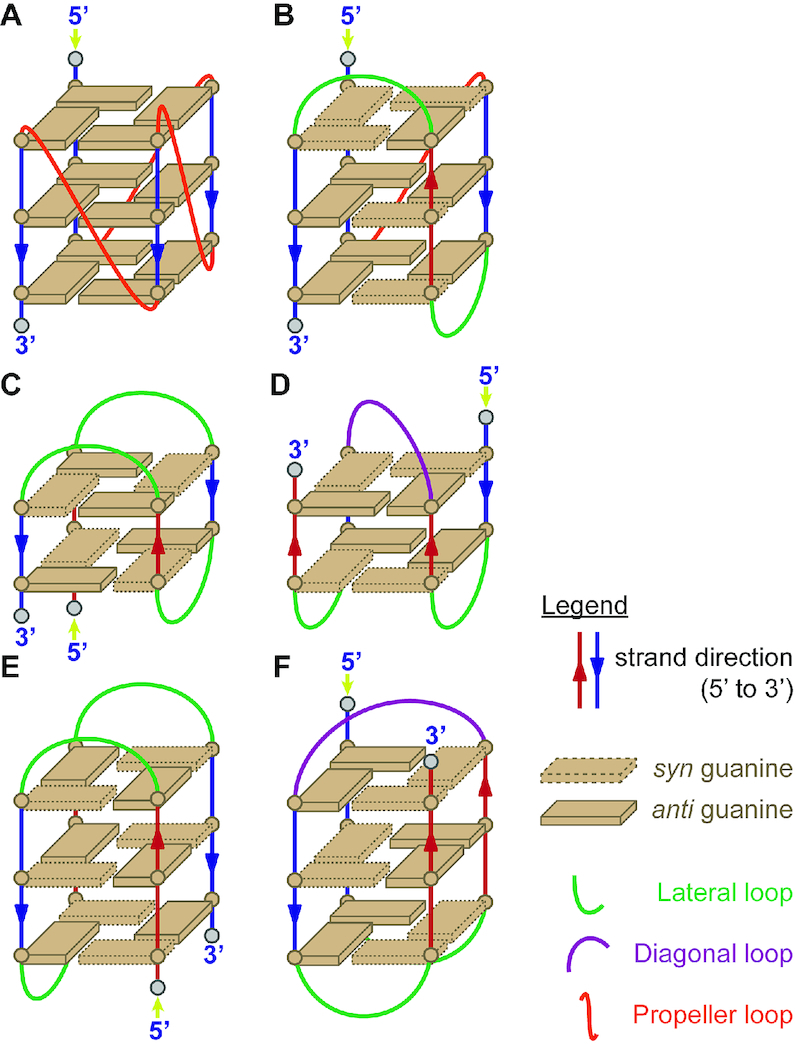
Schematic representation of different G4 topologies. (**A**) Parallel (type I stacking), (**B**) Hybrid (type II stacking), (**C**) Antiparallel 2-quartet (type III stacking with chair conformation), (**D**) Antiparallel 2-quartet (type III stacking with basket conformation), (**E**) Antiparallel 3-quartet (type II stacking with chair conformation), (**F**) Antiparallel 3-quartet (type III stacking with basket conformation, not represented in our database).

Our goal was to gather diverse topologies from the literature with NMR data in potassium. The published solution structures in potassium include many parallel topologies with type I base stacking (homo stacking), some hybrid topologies (in particular, among human telomeric sequences) with type II stacking (homo and hetero stacking), some antiparallel topologies with two G-quartets (type III stacking, hetero stacking), and one antiparallel topology with 3-quartet but a type II stacking (5YEY) (Figure 1A–E). There is no documented antiparallel topology with 3-quartets and a purely type III stacking in potassium; such structures are documented only in sodium (Figure 1F) (37).

The database was built using an in-house, open-source R package, *g4dbr* (https://github.com/EricLarG4/g4dbr). Specifically, the *g4db* function is dedicated to the processing, tidying, storing, visualization, and reporting of CD, ^1^H NMR, UV-melting and native MS data from oligonucleotide samples. Although developed for the G4 forming sequences characterized in this manuscript, *g4db* can be used with any nucleic acid sequence. The long-term goal is to provide open-source tools for the deposition of oligonucleotide biophysics data (raw and processed), while allowing for easy and versatile visualization and reporting.

In practice, users can employ the app to visualize a database previously generated by *g4db* in the *database* module, or visualize and process raw data, then import it into a new or existing database using the *importR* module (Supplementary Figure S169). To read raw data in *g4db*, it must be first pasted into a templated Excel file provided in the package. Several tools are included in the package for the automated processing of data, and can also be used outside of the database scope. The most notable are:

- Data filtering by oligonucleotide name, sequence, topology, buffer, cation, and selective writing to databases;
- Labeling of MS spectra performed from user-supplied species names, for which the expected *m/z* are calculated by the application;
- Calculation of molar extinction coefficients at 260 nm from the oligonucleotide sequence, using Equation (1). This can be used independently from g4db, using the *epsilon*.*calculator* function (included in the g4dbr package);
- Conversion of CD data in mdeg to molar extinction coefficient, using Equation (2);
- Determination of folded fraction versus temperature, and *T*_m_, from UV-melting data (*meltR* module), using Equation (3). This will be released as a standalone application, and its performance will be discussed in a separate publication;
- MS noise reduction by intensity filtering, which can be carried out independently with the *mass.diet* function (included in the package).

The processed data is consolidated into .Rda files, which can either be consulted in *g4db*, or can be loaded in base R for uses outside the application scope. In *g4db*, the data can be visualized with several customizable plots, and exported in reports in word, pdf, or HTML formats. The reports generated for the oligonucleotides characterized herein are collated in supporting information, and are accessible online as well (https://ericlarg4.github.io/G4_database.html). The use of g4db is described in supporting information (Supplementary Figures S170—S192) and the whole application can be found in the Zenodo repository (http://doi.org/10.5281/zenodo.4200176).

### Stability in ESI-MS conditions (1 mM KCl/100 mM TMAA) compared to ∼100 mM KCl

The melting temperatures are systematically lower in 1 mM KCl than in ∼100 mM KCl for all sequences studied (see Table 1). As a result, for some molecular systems the decrease is such that a fraction of the oligonucleotide is not folded at room temperature. In the ESI-MS spectra, this translates into the appearance of peaks with lower-than-predicted number of K^+^ ions bound. The number of K^+^ ions predicted to bind in the G-quadruplex core is *n-1*, with *n* the number of stacked G-quartets. However, whether all sites are fully filled depends on the K^+^ binding equilibrium constants and on the KCl concentration.

**Figure 2.**
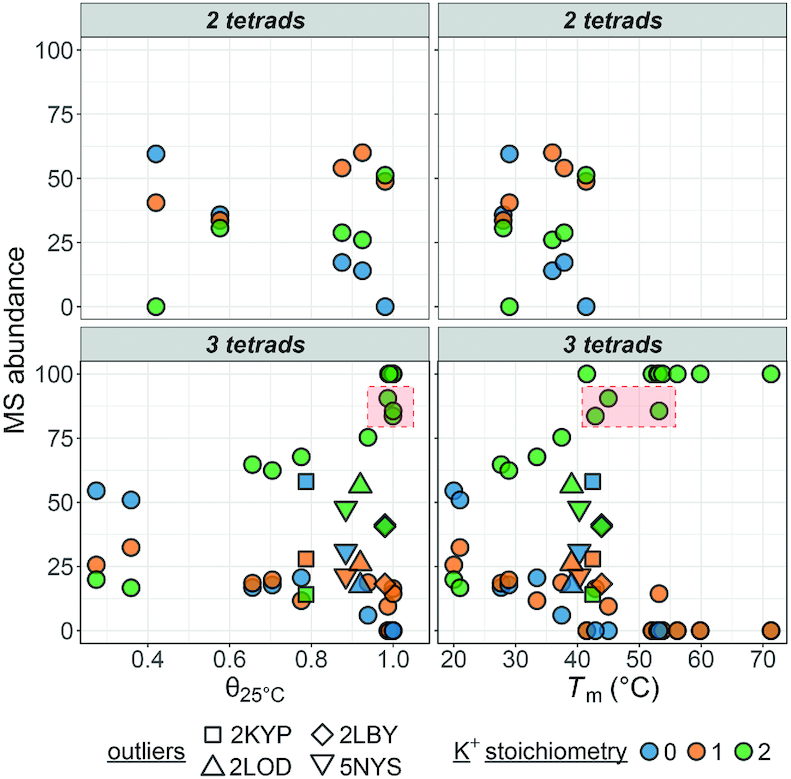
Species abundance (%) on the 5- charge state in native ESI-MS experiments against their folded fraction at 25°C (left) or melting temperature (right) determined by UV-melting. The oligonucleotides are grouped in panels by their number of G-quartets, and colored by their number of potassium adducts. The 2-K^+^ data of slight outliers (2M27, 2KPR and 5YEY) is framed in red, while four other outliers are distinguished by specific symbols.

Figure 2 shows that for the 3-quartet G4s, there is a good correlation between the melting temperature, the fraction folded at 25°C and the relative intensity of the 2-K^+^ complex in the ESI-MS conditions (values in Table 1, spectra in the supporting information). The 2-K^+^ abundance increases with the folded fraction or *T*_m_ for 3-quartet G4s. Usually, if *T*_m_ > 40°C (which corresponds to fraction folded at 25°C > 80%), there is no zero-potassium (non-folded) form in ESI-MS conditions. Slight outliers are 2M27, 2KPR and 5YEY where there is still ∼9%,14% and 16% 1-K^+^ complex although the fraction folded at 25°C is calculated as ∼99%, 100% and 100% respectively (square box in Figure 2, bottom panel). More obvious outliers are 2LBY, 5NYS, 2LOD and 2KYP, for which the combined abundance of 0-K^+^ and 1-K^+^ complex is > 40% even though according to the low-temperature baseline in UV-melting the structure seems fully formed at room temperature. These results show that under flat UV-melting baselines, several species with different number of tetrads can coexist, and that the transition can thus be due only to one of these species. This would merit further exploration by temperature-resolved mass spectrometry (38).

The trend is similar with 1-K^+^ (2-quartet) G4s (Figure 2, top panel), but fewer data points are available. Note that the interpretation of the K^+^ distribution in antiparallel 2-quartet structures is peculiar. The case of the 2-quartet 2KF8 was discussed in detail previously (where it was named 22GT) (22). In 2KF8, the main stoichiometry is 1K^+^, and the 2K^+^ complex reflects a second lower-affinity binding site between a quartet and a triplet, which upon stacking also changes the CD spectrum. We can thus imagine that in the 1-K^+^ complex the G-triple is not structured, while it is present in the 2-K^+^ complex. In contrast, sequences that do not involves stable base pairing (triplex or G–C–G–C) above the quartet at 1 mM K^+^ (2KM3, 5LQG) do not readily bind a second K^+^ ion.

### Solution structure in ESI-MS conditions (1 mM KCl/100 mM TMAA) compared to 100 mM K^+^

The ^1^H NMR spectra recorded in 1 mM KCl and 100 mM TMAA were compared to those published in the literature, and all matching peaks (in the imino region) are labeled according to the published base number assignment. In some cases, minor peaks were present (as indicated in the table), but note that in 100 mM K^+^ minor other conformations were also noticed for the wild type sequences. In such case we would still conclude that the main topology in ESI-MS conditions is the same as in the PDB.

We then compared the CD spectra obtained in ∼100 mM KCl and in 1 mM KCl + 100 mM TMAA, and noted when the shapes were identical, showed more homo-stacking (larger relative signal at 260 nm) or more hetero-stacking (larger relative signal at 290 nm). Parallel topologies show exclusively anti-anti guanine stacking (type I stacking) characterized in CD spectra by positive maximum at ∼265 nm and negative maxima at 245 nm. Hybrid G4s combine syn/anti and anti/syn with anti/anti stacking (type II stacking) in the topology which is represented by two positive peaks at 270 and 290 nm and 1 negative minimum at 245 nm. Antiparallel topologies usually display alternative stacking of syn/anti and anti/syn guanines (type III stacking) leading to the CD positive maxima at 290 nm and CD negative minima at 260 nm (39). Shape changes can hint at different structural populations, and when noticed, the confidence in having the same structure in ESI-MS conditions as in the published conditions was lower.

**Figure 3.**
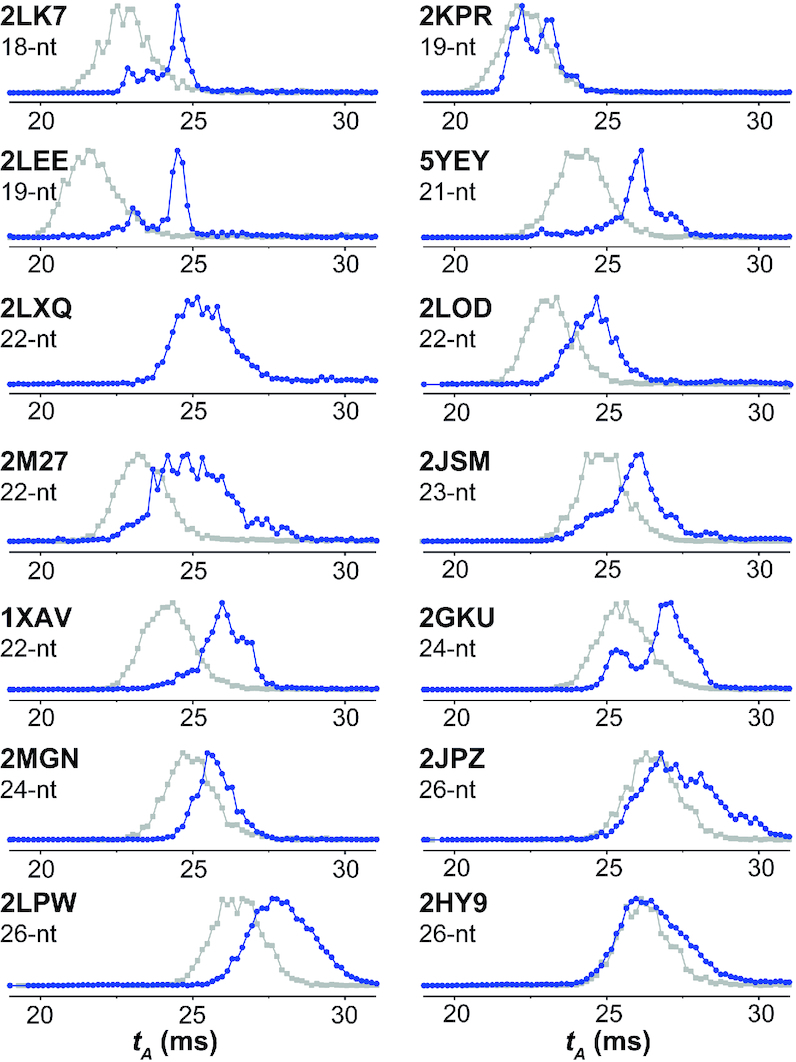
Electrospray ion mobility analysis of the folded form with 2 K^+^ (blue, recorded from 1 mM KCl and 100 mM TMAA) and the unfolded form with 0 K^+^ (gray, recorded from 100 mM TMAA) Total arrival time distributions (*t_A_*) of the 5– charge states for 0 K^+^ and 2K^+^ complex are shown. The left column gathers representative 3-quartet G4s with type-I stacking and the right column gathers all 3-quartet structures with type-II stacking.

We also analyzed the 2-K^+^ form of each sequence by ion mobility spectrometry, to observe if there is one or several conformational ensembles. Figure 3 shows the arrival time distribution measured for the 5- charge state of the 2-K^+^ complex (dark blue), compared to the 0-K^+^ complex (light grey). In all cases the peak shape differs, indicating a memory of the presence of the G-quadruplex in the 2-K^+^ complexes. However, the interpretation in terms of solution structure is not straightforward, and will be described in more detail in a separate publication. Briefly, the collision cross section values extracted from the arrival time distributions are systematically smaller than those computed from the solution (PDB) coordinates. Gas-phase rearrangements always induce compaction of the 5- charge states (40,41). Sequences with longer loops or overhangs lead to broad ion mobility peaks, due to the various ways the loops can rearrange in the gas-phase. Consequently, sharp peaks are only observed for short sequences (< 20-nt), or 21–24-nt sequences wherein loops are specifically locked in by base pairs or triples (for example, 5YEY, 2MGN, or 2GKU), while all 26-nt G-quadruplexes showed broad peaks. When several peaks are observed, it can either mean that a second topology (different G-quartet arrangement) coexists (as described for 2GKU (22,42) or 2JSM (22,43), or that a fraction of the population has not all its loop bases locked in at room temperature. In several cases with a second ion mobility peak, minor imino proton peaks are also visible in ^1^H NMR (2GKU, 2JSM, 2HY9, 2JPZ, 2LOD), but not always (for example the origin of the two mobility peaks for 2KPR is not elucidated). For these reasons, and contrary to our expectations, the ion mobility data was thus found of limited use in the context of validation of the preservation of solution structures in ESI-MS conditions.

In Table 1 we rated each sequence. Two stars (**) means that the folding is >90% complete in mainly the same topology as formed by NMR. One star (*) means that either the folding is incomplete, or that there are doubts that the main topologies formed are the same in 1 and ∼100 mM KCl. Sequences with incomplete folding can be problematic for ligand screening by ESI-MS, because ligands that bind to the folded fraction also have to displace the folding equilibrium, and therefore the apparent binding affinity is the convoluted result of folding and binding equilibria. Nevertheless, it is still possible that the folded fraction has the same structure as the ∼100 mM K^+^ (NMR) structure, and that these sequences might thus still be of use for structural or specificity studies. Below we discuss the behavior of different G-quadruplex families in more detail.

#### Human telomeric sequences and variants

**Figure 4.**
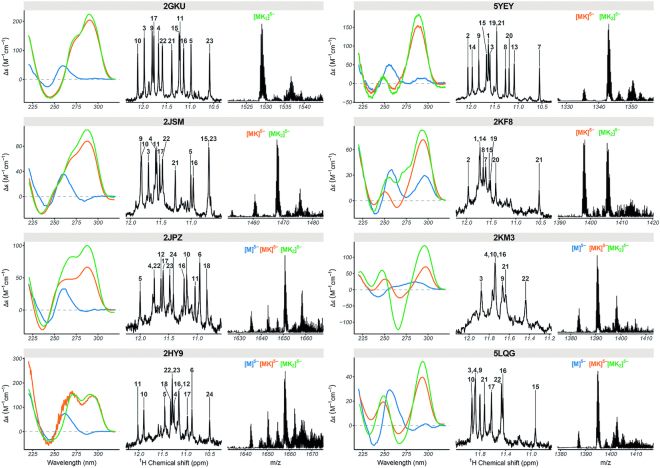
Circular dichroism (left; 10 μM DNA in blue: 100 mM TMAA, orange: 100 mM TMAA + 1 mM KCl, green: potassium phosphate + KCl), ^1^H-NMR (center; 100 μM DNA in 100 mM TMAA + 1 mM KCl), and native ESI-MS (right; 10 μM DNA in 100 mM TMAA + 1 mM KCl; M: monomer, K: potassium) data of selected human telomeric quadruplex-forming oligonucleotides.

Figure 4 gathers the CD, NMR and K^+^ adduct distribution of eight models with known structures. This group includes one 3-quartet antiparallel topology (5YEY), four 3-quartet hybrid topologies (2JSM, 2GKU, 2HY9 and 2JPZ), and three 2-quartet antiparallel topologies (2KF8, 2KM3, 5LQG). For all hybrid topologies, we observe the 3-quartet [M+2K]^*n*−^ complex as the major species in 1 mM K^+^. However, for hybrid G4 sequences (except 2GKU) we observe a substantial population of 2-quartet [M+1K]^*n*−^ and non-folded species [M+0K]^*n*−^, indicating an incomplete folding in 1 mM KCl (2JPZ, 2HY9 and 2JSM) (Figure 4). The subpopulation of minor species is also evident from the unassigned peaks in the imino region of ^1^H-NMR spectra. For hybrid-1 telomeric G4s, the preferred sequences for ligand screening should be 2GKU and 2JSM (the first is more stable and less polymorphic, but the overhangs are not fully faithful to telomeric repeats). For hybrid-2 we have only one sequence representative, 2JPZ, which is incompletely folded (θ_25_ = 0.65). Therefore, it would be desirable to find another suitable hybrid-2 telomeric sequences, stable enough in ESI-MS conditions for ligand screening. Note that there is also evidence of coexistence of hybrid-1 and hybrid-2 topologies (42,43), in line with ion mobility results showing several peaks (see Figure 3 for 2JSM; 2GKU, 2JPZ and the deformed peak for 2HY9).

In antiparallel sequences we have two antiparallel 2-quartet sequences with basket topology (2KF8, 5LQG) and one chair topology (2KM3). For 2KM3 and 5LQG, [M+1K]^*n*−^ complexes predominate in ESI MS while for 2KF8 [M+1K]^*n*−^ and [M+2K]^*n*−^ peaks coexist (Figure 4). The second K^+^ binding site is enabled by the formation of guanine triplet composed of G9, G13, and G21 in the diagonal loop . 5LQG can form two different antiparallel basket-type G4s depending upon K^+^ concentration and pH as shown previously (44), and is incompletely folded, which makes it unsuitable for ligand screening in MS. Finally, for 5YEY we observe a major population of [M+2K]^*n*−^ and mostly one conformation according to ^1^H-NMR spectra. In summary, for antiparallel sequences, the best sequences for ligand screening are 2KF8 (antiparallel 2-quartet, basket) and 5YEY (antiparallel 3-quartet, chair). 2KM3 is the only antiparallel 2-quartet with a chair conformation, but is incompletely folded (θ_25_ = 0.87).

Two frequently used polymorphic sequences (21G, 22AG) are also included in the database. These sequences show mixed conformational topology from CD and broad imino proton signal in ^1^H NMR spectra in the ESI-MS buffer as well. Their mass spectra show both [M+1K]^*n*−^ and [M+2K]^*n*−^ (*n* = 4, 5, 6) stoichiometries. Their ion binding distribution and melting temperatures are very similar to those of 2KF8, indicating significant amounts of 2-quartet topologies in the MS-compatible conditions. Ion mobility spectrometry results are also similar to those of the 2-quartet models (see supporting information Figure S193).

#### Parallel G-quadruplexes

**Figure 5.**
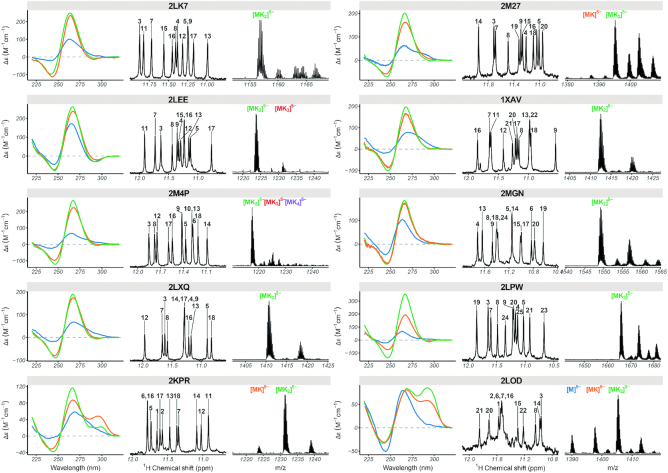
Circular dichroism (left; 10 μM DNA in blue: 100 mM TMAA, orange: 100 mM TMAA + 1 mM KCl, green: potassium phosphate + KCl), ^1^H NMR (center; 100 μM DNA in 100 mM TMAA + 1 mM KCl), and native ESI-MS (right; 10 μM DNA in 100 mM TMAA + 1 mM KCl; M: monomer, K: potassium) data of selected parallel and hybrid quadruplex-forming oligonucleotides.

A second group of non-telomeric (mainly promoter sequences) with validated folds are parallel-stranded with type I stacking. Eight sequences with length ranging from 18 to 26 nucleotides are fully folded (>90%) in the ESI-MS conditions. These include two artificial constructs (PDB: 2LK7, 2M4P), two c-myc promoter variants (PDB: 1XAV, 2A5P/2MGN), as well as N-myc (PDB: 2LEE), VEGF (PDB: 2M27), Neisseria gonorrhoeae pilE promoters (PDB: 2LXQ) and the CEB25 human minisatellite (PDB: 2LPW) (Figure 5). Another three sequences (2LBY, another c-myc promoter variant; 2O3M, the c-kit promoter; 5NYS, an artificial construct) also form the same parallel structure as reported by NMR, but with a significant fraction unfolded (0K). These three sequences should not be used for testing ligand preference to parallel versus other topologies, but can be used with cautious interpretation of the apparent binding constants if one is interested in these specific structures. Finally, the c-kit2 (PDB: 2KYP), HIV-1 LTR (PDB: 2N4Y), and KRAS (PDB: 5I2V) do not fold in the desired topology in 1 mM KCl.

#### Other model structures

To have a few other topologies adequate for ligand screening in ESI-MS, we tested two other 3-quartet hybrid structures (2KPR and 2LOD) and two 2-quartet antiparallel structures (6GZN, HIV-PRO1). The two hybrid folds present both similarities (NMR) and differences (CD) between 100 mM and 1 mM KCl conditions (Figure 5). Both sequences have a major population of [M+2K]^*n*−^, but also [M+1K]^*n*−^ as a minor species in 1 mM K^+^. Therefore, the confidence rating is low (‘*’) compared to the representatives of type I folding. The two antiparallel structures did not form the same fold in ESI-MS conditions as evident from ^1^H NMR, CD and ESI-MS.

#### Recommendations for including further sequences for native MS screening

In order to short-list further sequences for biophysical and ligand screening studies by native mass spectrometry, the following points must be considered:

1. Pre-select sequences that can potentially be of interest due to their origin or biological function, based on the following factors:
  - A high-resolution structure is available (probably by solution NMR in K^+^), obtained from a non-modified sequence.
  - The structure does reflect a single or predominant/major conformation in solution.
  - Sequences of the same length but different topology can always be an interesting point to screen for a similar group of ligands to observe ‘selectivity’.
2. Perform the experiments and the data processing in a consistent manner to ensure the validity of comparison across techniques and oligonucleotides, and minimize the variability of results.
  - Establish and use standard protocols in each experiment type for all sequences concerned.
  - Where applicable, normalize data to streamline comparisons. For instance, CD data obtained for different oligonucleotide concentrations and/or cuvette path lengths can be more easily compared if the data is converted to Δϵ (Equation 2).
  - As far as possible, eliminate human biases from the data processing, by e.g. automation. For instance, the ‘manual’ baseline subtraction of UV-melting data is notoriously imprecise (34), and was automated herein (Equation 3).
3. To compare with the high-resolution structure (NMR derived) in high K^+^ containing buffer, it is necessary to perform additional solution spectroscopic experiments (CD, UV melting) in both conditions (1 mM K^+^ and ≥1 mM K^+^). Finally, by comparing imino proton signals in ^1^H NMR with the published assignment, one can conclude whether a particular sequence retains its same tetrad arrangement or not. ^1^H NMR can also give some information on the formation of triads/base pairs apart from the quartet formation.
4. Since many G-quadruplexes are polymorphic, at 1 mM K^+^ one often finds minor populations for other misfolded species/alternative conformations. Here we focused on comparing the major species observed in ESI-MS (3 quartet for type I and II and 2 quartets for type-III folding) with the major species in ^1^H-NMR. Ion mobility spectrometry interpretation is still subject to caution. A single narrow peak indicates one main well-locked conformation, but based on the current understanding of gas-phase rearrangements, interpreting complex mobility patterns in terms of solution conformational ensembles is not recommended. In-depth structural analysis for each of the conformers for every sequence in native MS buffer by solution NMR is preferred but was, however, beyond the scope of the manuscript. Overall, we recommend to discard sequences for which a significant amount of unfolded folded species is found (^1^H NMR not matching with literature, different CD profile, θ_25_ << 1 in UV melting, and high abundance of the 0-K^+^ complex in ESI-MS).
5. Consolidate the data in a way that provides the community with open and easy access to raw and processed data, as well as all necessary contextual elements (e.g. oligonucleotide sequence, extinction coefficient, and concentration, buffer and cation nature and concentration, literature references). Here we developed a database and software suite to automatize the gathering, data treatment, storage, and reporting in a completely open way.

## DISCUSSION

We here listed sequences with different topology and validated fold (** in Table 1) to be used as targets for ligand screening by native MS. We note however that the database is biased towards parallel 3-quartet structures because (1) they are over-represented among the intramolecular structures solved to date in potassium and (2) they are typically more stable and less polymorphic, and thus more likely to be preserved in 1 mM KCl. For non-parallel structures, the best models for ESI-MS screening are variants of the human telomeric sequence, with the known caveat that due to inherent polymorphism, ligand-induced conformational changes can occur even for sequences that have one very predominant fold in absence of ligand (28,45,46).

Finding suitable candidates with intramolecular type II & type III folding among non-telomeric sequences for MS-based ligand screening in 1 mM K^+^ remains challenging because there are not many such structures solved already in ∼100 mM K^+^. Note that for now we limited our database to intramolecular G-quadruplex forming sequences with all-natural nucleotides. Antiparallel structures with more than two quartets are rare in potassium. Among bimolecular G quadruplex sequences, previous results showed that (12TAG)_2_ and (G_4_T_4_G_3_)_2_ are not stable enough in 1 mM K^+^ (46,47), but (dG_4_T_4_G_4_)_2_ and (dG_4_T_3_G_4_)_2_ shows [M+3K]^*n*−^ as major peaks corresponding to 4 quartet antiparallel conformation while (G_3_T_4_G_4_)_2_ form (M+2K)^*n*−^ in 1 mM KCl. Whether these structures are the same as in solution NMR studies (24,46,48,49) remains to be established. The other possibility for expanding the conformational space is to use ammonium acetate (NH_4_OAc) when there is sufficient evidence of conformation and NH_4_^+^ binding from solution NMR studies (24). We will continue to update the database and, in particular, we will incorporate more sequences with unusual quadruplex folding (e.g. left-handed quadruplex, quadruplex-duplex hybrid) and bimolecular/tetra molecular quadruplexes in our online database to have a robust library of G4s.

## DATA AVAILABILITY

The reports generated for the oligonucleotides characterized herein are accessible online as well (https://ericlarg4.github.io/G4_database.html). The whole application is deposited to Zenodo repository (DOI: 10.5281/zenodo.4200176).

## Supplementary Material

gkab039_Supplemental_File

## References

[1] Saenger W. Principles of Nucleic Acid Structure. 1984; NY Springer Science & Business Media.

[2] Choi J. , Majima T. Conformational changes of non-B DNA. Chem. Soc. Rev. 2011; 40:5893–5909. 21901191 10.1039/c1cs15153c

[3] Rhodes D. , Lipps H.J. G-quadruplexes and their regulatory roles in biology. Nucleic. Acids. Res. 2015; 43:8627–8637. 26350216 10.1093/nar/gkv862 PMC4605312

[4] Huppert J.L. Four-stranded nucleic acids: structure, function and targeting of G-quadruplexes. Chem. Soc. Rev. 2008; 37:1375–1384. 18568163 10.1039/b702491f

[5] Cahoon L.A. , Seifert H.S. An alternative DNA structure is necessary for pilin antigenic variation in Neisseria gonorrhoeae. Science. 2009; 325:764–767. 19661435 10.1126/science.1175653 PMC2803317

[6] Collie G.W. , Parkinson G.N. The application of DNA and RNA G-quadruplexes to therapeutic medicines. Chem. Soc. Rev. 2011; 40:5867–5892. 21789296 10.1039/c1cs15067g

[7] Mergny J.-L. , Hélène C. G-quadruplex DNA: a target for drug design. Nat. Med. 1998; 4:1366–1367. 9846570 10.1038/3949

[8] Balasubramanian S. , Neidle S. G-quadruplex nucleic acids as therapeutic targets. Curr. Opin. Chem. Biol. 2009; 13:345–353. 19515602 10.1016/j.cbpa.2009.04.637 PMC2726962

[9] Yatsunyk L.A. , Mendoza O., Mergny J.-L. ‘Nano-oddities’: unusual nucleic acid assemblies for DNA-based nanostructures and nanodevices. Acc. Chem. Res. 2014; 47:1836–1844. 24871086 10.1021/ar500063x

[10] Mergny J.-L. , Sen D DNA quadruple helices in nanotechnology. Chem. Rev. 2019; 119:6290–6325. 30605316 10.1021/acs.chemrev.8b00629

[11] Bates P.J. , Reyes-Reyes E.M., Malik M.T., Murphy E.M., O’Toole M.G., Trent J.O. G-quadruplex oligonucleotide AS1411 as a cancer-targeting agent: Uses and mechanisms General subjects. Biochim. Biophys. Acta Gen. Subj. 2017; 1861:1414–1428. 28007579 10.1016/j.bbagen.2016.12.015

[12] Carvalho J. , Paiva A., Campello M.P.C., Paulo A., Mergny J.-L., Salgado G.F., Queiroz J.A., Cruz C Aptamer-based targeted Delivery of a G-quadruplex Ligand in Cervical Cancer Cells. Sci. Rep. 2019; 9:7945. 31138870 10.1038/s41598-019-44388-9 PMC6538641

[13] Neidle S. , Parkinson G.N. The structure of telomeric DNA. Curr. Opin. Struct. Biol. 2003; 13:275–283. 12831878 10.1016/s0959-440x(03)00072-1

[14] Webba da Silva M. Geometric formalism for DNA quadruplex folding. Chem. Eur. J. 2007; 13:9738–9745. 17972263 10.1002/chem.200701255

[15] Karsisiotis A.I. , O’Kane C., da Silva M.W. DNA quadruplex folding formalism–a tutorial on quadruplex topologies. Methods. 2013; 64:28–35. 23791747 10.1016/j.ymeth.2013.06.004

[16] Neidle S. Human telomeric G-quadruplex: The current status of telomeric G-quadruplexes as therapeutic targets in human cancer. FEBS J. 2010; 277:1118–1125. 19951354 10.1111/j.1742-4658.2009.07463.x

[17] Phan A.T. Human telomeric G-quadruplex: structures of DNA and RNA sequences. FEBS J. 2010; 277:1107–1117. 19951353 10.1111/j.1742-4658.2009.07464.x

[18] Largy E. , Mergny J.-L., Gabelica V. The Alkali Metal Ions: Their Role for Life. 2016; Springer 203–258. 10.1007/978-3-319-21756-7_7 26860303

[19] Largy E. , Marchand A., Amrane S., Gabelica V., Mergny J.-L. Quadruplex turncoats: cation-dependent folding and stability of quadruplex-DNA double switches. J. Am. Chem. Soc. 2016; 138:2780–2792. 26837276 10.1021/jacs.5b13130

[20] Parkinson G.N. , Lee M.P., Neidle S. Crystal structure of parallel quadruplexes from human telomeric DNA. Nature. 2002; 417:876–880. 12050675 10.1038/nature755

[21] Wang Y. , Patel D.J. Solution structure of the human telomeric repeat d[AG3(T2AG3)3] G-tetraplex. Structure. 1993; 1:263–282. 8081740 10.1016/0969-2126(93)90015-9

[22] Marchand A. , Gabelica V. Folding and misfolding pathways of G-quadruplex DNA. Nucleic Acids Res. 2016; 44:10999–11012. 27924036 10.1093/nar/gkw970 PMC5159560

[23] Marchand A. , Gabelica V. Native electrospray mass spectrometry of DNA G-quadruplexes in potassium solution. J. Am. Soc. Mass. Spectrom. 2014; 25:1146–1154. 24781455 10.1007/s13361-014-0890-3 PMC4055847

[24] Balthasart F. , Plavec J., Gabelica V. Ammonium ion binding to DNA G-quadruplexes: do electrospray mass spectra faithfully reflect the solution-phase species?. J. Am. Soc. Mass. Spectrom. 2013; 24:1–8. 23132414 10.1007/s13361-013-0649-2 PMC5110665

[25] Marchand A. , Ferreira R., Tateishi-Karimata H., Miyoshi D., Sugimoto N., Gabelica V. Sequence and solvent effects on telomeric DNA bimolecular G-quadruplex folding kinetics. J. Phys. Chem. B. 2013; 117:12391–12401. 23978125 10.1021/jp406857s

[26] Rosu F. , Gabelica V., Houssier C., Colson P., Pauw E.D. Triplex and quadruplex DNA structures studied by electrospray mass spectrometry. Rapid Commun. Mass Spectrom. 2002; 16:1729–1736. 12207360 10.1002/rcm.778

[27] Gros J. , Rosu F., Amrane S., De Cian A., Gabelica V., Lacroix L., Mergny J.-L. Guanines are a quartet's best friend: impact of base substitutions on the kinetics and stability of tetramolecular quadruplexes. Nucleic. Acids. Res. 2007; 35:3064–3075. 17452368 10.1093/nar/gkm111 PMC1888817

[28] Marchand A. , Granzhan A., Iida K., Tsushima Y., Ma Y., Nagasawa K., Teulade-Fichou M.-P., Gabelica V. Ligand-induced conformational changes with cation ejection upon binding to human telomeric DNA G-quadruplexes. J. Am. Chem. Soc. 2015; 137:750–756. 25525863 10.1021/ja5099403

[29] Marchand A. , Rosu F., Zenobi R., Gabelica V. Thermal denaturation of DNA G-quadruplexes and their complexes with ligands: thermodynamic analysis of the multiple states revealed by mass spectrometry. J. Am. Chem. Soc. 2018; 140:12553–12565. 30183275 10.1021/jacs.8b07302

[30] Scalabrin M. , Palumbo M., Richter S.N. Highly improved electrospray ionization-mass spectrometry detection of G-quadruplex-folded oligonucleotides and their complexes with small molecules. Anal. Chem. 2017; 89:8632–8637. 28787153 10.1021/acs.analchem.7b01282 PMC5588092

[31] Ferreira R. , Marchand A., Gabelica V. Mass spectrometry and ion mobility spectrometry of G-quadruplexes. A study of solvent effects on dimer formation and structural transitions in the telomeric DNA sequence d (TAGGGTTAGGGT). Methods. 2012; 57:56–63. 22465284 10.1016/j.ymeth.2012.03.021

[32] Cantor C.R. , Warshaw M.M., Shapiro H. Oligonucleotide interactions. III. Circular dichroism studies of the conformation of deoxyoligonucleolides. Biopolymers. 1970; 9:1059–1077. 5449435 10.1002/bip.1970.360090909

[33] Tataurov A.V. , You Y., Owczarzy R. Predicting ultraviolet spectrum of single stranded and double stranded deoxyribonucleic acids. Biophys. Chem. 2008; 133:66–70. 18201813 10.1016/j.bpc.2007.12.004

[34] Mergny J.-L. , Lacroix L. Analysis of thermal melting curves. Oligonucleotides. 2003; 13:515–537. 15025917 10.1089/154545703322860825

[35] Plateau P. , Gueron M. Exchangeable proton NMR without base-line distorsion, using new strong-pulse sequences. J. Am. Chem. Soc. 1982; 104:7310–7311.

[36] Gabelica V. , Livet S., Rosu F. Optimizing native ion mobility Q-TOF in helium and nitrogen for very fragile noncovalent structures. J. Am. Soc. Mass. Spectrom. 2018; 29:2189–2198. 30047072 10.1007/s13361-018-2029-4

[37] Dvorkin S.A. , Karsisiotis A.I., da Silva M.W. Encoding canonical DNA quadruplex structure. Sci. Adv. 2018; 4:eaat3007. 30182059 10.1126/sciadv.aat3007 PMC6118410

[38] Marchand A. , Rosu F., Zenobi R., Gabelica V. Thermal denaturation of DNA G-quadruplexes and their complexes with ligands: thermodynamic analysis of the multiple states revealed by mass spectrometry. J. Am. Chem. Soc. 2018; 140:12553–12565. 30183275 10.1021/jacs.8b07302

[39] Karsisiotis A.I. , Hessari N.M., Novellino E., Spada G.P., Randazzo A., Webba da Silva M. Topological characterization of nucleic acid G-quadruplexes by UV absorption and circular dichroism. Angew. Chem. Int. Ed. 2011; 123:10833–10836. 10.1002/anie.201105193 21928459

[40] D’Atri V. , Gabelica V. DNA and RNA telomeric G-quadruplexes: what topology features can be inferred from ion mobility mass spectrometry?. Analyst. 2019; 144:6074–6088. 31528871 10.1039/c9an01216h

[41] Porrini M. , Rosu F., Rabin C., Darre L., Gomez H., Orozco M., Gabelica V. Compaction of duplex nucleic acids upon native electrospray mass spectrometry. ACS Cent. Sci. 2017; 3:454–461. 28573208 10.1021/acscentsci.7b00084 PMC5445532

[42] Bessi I. , Jonker H.R., Richter C., Schwalbe H. Involvement of long-lived intermediate states in the complex folding pathway of the human telomeric G-quadruplex. Angew. Chem. Int. Ed. 2015; 54:8444–8448. 10.1002/anie.201502286 26036989

[43] Frelih T. , Wang B., Plavec J., Šket P. Pre-folded structures govern folding pathways of human telomeric G-quadruplexes. Nucleic Acids Res. 2020; 48:2189–2197. 31950178 10.1093/nar/gkz1235 PMC7038944

[44] Galer P. , Wang B., Šket P., Plavec J. Reversible pH switch of two-quartet G-quadruplexes formed by human telomere. Angew. Chem. 2016; 128:2033–2037. 10.1002/anie.201507569 26836334

[45] Marchand A. , Strzelecka D., Gabelica V. Selective and cooperative ligand binding to antiparallel human telomeric DNA G-quadruplexes. Chemistry. 2016; 22:9551–9555. 27168452 10.1002/chem.201601937

[46] Lecours M.J. , Marchand A., Anwar A., Guetta C., Hopkins W.S., Gabelica V. What stoichiometries determined by mass spectrometry reveal about the ligand binding mode to G-quadruplex nucleic acids. Biochim. Biophys. Acta Gen. Subj. 2017; 1861:1353–1361. 28087374 10.1016/j.bbagen.2017.01.010

[47] Phan A.T. , Patel D.J. Two-repeat human telomeric d(TAGGGTTAGGGT) sequence forms interconverting parallel and antiparallel G-quadruplexes in solution: distinct topologies, thermodynamic properties, and folding/unfolding kinetics. J. Am. Chem. Soc. 2003; 125:15021–15027. 14653736 10.1021/ja037616j PMC4693644

[48] Šket P. , Črnugelj M., Plavec J. d (G3T4G4) forms unusual dimeric G-quadruplex structure with the same general fold in the presence of K+, Na+ or NH4+ ions. Biorg. Med. Chem. 2004; 12:5735–5744. 10.1016/j.bmc.2004.08.009 15498650

[49] Schultze P. , Smith F.W., Feigon J. Refined solution structure of the dimeric quadruplex formed from the Oxytricha telomeric oligonucleotide d (GGGGTTTTGGGG). Structure. 1994; 2:221–233. 8069635 10.1016/s0969-2126(00)00023-x

[50] Luu K. , Phan A., Kuryavyi V., Lacroix L., Patel D Monomeric Human Telomere DNA Tetraplex with 3+ 1 Strand Fold Topology, Two Edgewise Loops and Double-Chain Reversal Loop, NMR, 12 Structures. J. Am. Chem. Soc. 2006; 128:9963–9970. 16866556 10.1021/ja062791w PMC4692383

[51] Phan A.T. , Kuryavyi V., Luu K.N., Patel D.J Structure of two intramolecular G-quadruplexes formed by natural human telomere sequences in K+ solution. Nucleic. Acids. Res. 2007; 35:6517–6525. 17895279 10.1093/nar/gkm706 PMC2095816

[52] Dai J. , Carver M., Punchihewa C., Jones R.A., Yang D Structure of the Hybrid-2 type intramolecular human telomeric G-quadruplex in K+ solution: insights into structure polymorphism of the human telomeric sequence. Nucleic Acids Res. 2007; 35:4927–4940. 17626043 10.1093/nar/gkm522 PMC1976458

[53] Dai J. , Punchihewa C., Ambrus A., Chen D., Jones R.A., Yang D. Structure of the intramolecular human telomeric G-quadruplex in potassium solution: a novel adenine triple formation. Nucleic Acids Res. 2007; 35:2440–2450. 17395643 10.1093/nar/gkm009 PMC1874667

[54] Liu C. , Zhou B., Geng Y., Tam D.Y., Feng R., Miao H., Xu N., Shi X., You Y., Hong Y. et al. A chair-type G-quadruplex structure formed by a human telomeric variant DNA in K+ solution. Chem. Sci. 2019; 10:218–226. 30713633 10.1039/c8sc03813a PMC6330691

[55] Lim K.W. , Amrane S., Bouaziz S., Xu W., Mu Y., Patel D.J., Luu K.N., Phan A.T. Structure of the human telomere in K+ solution: a stable basket-type G-quadruplex with only two G-tetrad layers. J. Am. Chem. Soc. 2009; 131:4301–4309. 19271707 10.1021/ja807503g PMC2662591

[56] Lim K.W. , Alberti P., Guedin A., Lacroix L., Riou J.-F., Royle N.J., Mergny J.-L., Phan A.T.n. Sequence variant (CTAGGG)n in the human telomere favors a G-quadruplex structure containing a G· C· G· C tetrad. Nucleic Acids Res. 2009; 37:6239–6248. 19692585 10.1093/nar/gkp630 PMC2764449

[57] Micco M. , Collie G.W., Dale A.G., Ohnmacht S.A., Pazitna I., Gunaratnam M., Reszka A.P., Neidle S. Structure-based design and evaluation of naphthalene diimide G-quadruplex ligands as telomere targeting agents in pancreatic cancer cells. J. Med. Chem. 2013; 56:2959–2974. 23514618 10.1021/jm301899y

[58] Do N.Q. , Phan A.T. Monomer–dimer equilibrium for the 5′–5′ stacking of propeller-type parallel-stranded G-quadruplexes: NMR structural study. Chem. Eur. J. 2012; 18:14752. 23019076 10.1002/chem.201103295

[59] Trajkovski M. , Webba da Silva M., Plavec J. Unique structural features of interconverting monomeric and dimeric G-quadruplexes adopted by a sequence from the intron of the N-myc gene. J. Am. Chem. Soc. 2012; 134:4132–4141. 22303871 10.1021/ja208483v

[60] Mukundan V.T. , Phan A.T. Bulges in G-quadruplexes: broadening the definition of G-quadruplex-forming sequences. J. Am. Chem. Soc. 2013; 135:5017–5028. 23521617 10.1021/ja310251r

[61] Kuryavyi V. , Cahoon L.A., Seifert H.S., Patel D.J. RecA-binding pilE G4 sequence essential for pilin antigenic variation forms monomeric and 5′ end-stacked dimeric parallel G-quadruplexes. Structure. 2012; 20:2090–2102. 23085077 10.1016/j.str.2012.09.013 PMC3845372

[62] Agrawal P. , Hatzakis E., Guo K., Carver M., Yang D Solution structure of the major G-quadruplex formed in the human VEGF promoter in K+: insights into loop interactions of the parallel G-quadruplexes. Nucleic Acids Res. 2013; 41:10584–10592. 24005038 10.1093/nar/gkt784 PMC3905851

[63] Ambrus A. , Chen D., Dai J.X., Jones R.A., Yang D. Solution structure of the biologically relevant G-quadruplex element in the human c-MYC promoter. implications for G-quadruplex stabilization. Biochemistry. 2005; 44:2048–2058. 15697230 10.1021/bi048242p

[64] Phan A.T. , Kuryavyi V., Gaw H.Y., Patel D.J. Small-molecule interaction with a five-guanine-tract G-quadruplex structure from the human MYC promoter. Nat. Chem. Biol. 2005; 1:167–173. 16408022 10.1038/nchembio723 PMC4690526

[65] Chung W.J. , Heddi B., Hamon F., Teulade-Fichou M.P., Phan A.T. Solution Structure of a G-quadruplex Bound to the Bisquinolinium Compound Phen-DC3. Angew. Chem. Int. Ed. 2014; 53:999–1002. 10.1002/anie.201308063 24356977

[66] Amrane S. , Adrian M., Heddi B., Serero A., Nicolas A., Mergny J.-L., Phan A.T. Formation of pearl-necklace monomorphic G-quadruplexes in the human CEB25 minisatellite. J. Am. Chem. Soc. 2012; 134:5807–5816. 22376028 10.1021/ja208993r

[67] Mathad R.I. , Hatzakis E., Dai J., Yang D. c-MYC promoter G-quadruplex formed at the 5′-end of NHE III 1 element: insights into biological relevance and parallel-stranded G-quadruplex stability. Nucleic Acids Res. 2011; 39:9023–9033. 21795379 10.1093/nar/gkr612 PMC3203601

[68] Phan A.T. , Kuryavyi V., Burge S., Neidle S., Patel D.J. Structure of an unprecedented G-quadruplex scaffold in the human c-kit promoter. J. Am. Chem. Soc. 2007; 129:4386–4392. 17362008 10.1021/ja068739h PMC4693632

[69] Trajkovski M. , Endoh T., Tateishi-Karimata H., Ohyama T., Tanaka S., Plavec J., Sugimoto N. Pursuing origins of (poly) ethylene glycol-induced G-quadruplex structural modulations. Nucleic Acids Res. 2018; 46:4301–4315. 29648656 10.1093/nar/gky250 PMC5934638

[70] Kuryavyi V. , Phan A.T., Patel D.J. Solution structures of all parallel-stranded monomeric and dimeric G-quadruplex scaffolds of the human c-kit2 promoter. Nucleic Acids Res. 2010; 38:6757–6773. 20566478 10.1093/nar/gkq558 PMC2965254

[71] De Nicola B. , Lech C.J., Heddi B., Regmi S., Frasson I., Perrone R., Richter S.N., Phan A.T. Structure and possible function of a G-quadruplex in the long terminal repeat of the proviral HIV-1 genome. Nucleic Acids Res. 2016; 44:6442–6451. 27298260 10.1093/nar/gkw432 PMC5291261

[72] Kerkour A. , Marquevielle J., Ivashchenko S., Yatsunyk L.A., Mergny J.-L., Salgado G.F. High-resolution three-dimensional NMR structure of the KRAS proto-oncogene promoter reveals key features of a G-quadruplex involved in transcriptional regulation. J. Biol. Chem. 2017; 292:8082–8091. 28330874 10.1074/jbc.M117.781906 PMC5427283

[73] Kuryavyi V. , Patel D.J. Solution structure of a unique G-quadruplex scaffold adopted by a guanosine-rich human intronic sequence. Structure. 2010; 18:73–82. 20152154 10.1016/j.str.2009.10.015 PMC3381514

[74] Marušič M. , Šket P., Bauer L., Viglasky V., Plavec J. Solution-state structure of an intramolecular G-quadruplex with propeller, diagonal and edgewise loops. Nucleic Acids Res. 2012; 40:6946–6956. 22532609 10.1093/nar/gks329 PMC3413137

[75] Amrane S. , Kerkour A., Bedrat A., Vialet B., Andreola M.-L., Mergny J.-L. Topology of a DNA G-quadruplex structure formed in the HIV-1 promoter: a potential target for anti-HIV drug development. J. Am. Chem. Soc. 2014; 136:5249–5252. 24649937 10.1021/ja501500c

[76] Lenarčič Živković M. , Rozman J., Plavec J. Adenine-driven structural switch from a two-to three-quartet DNA G-quadruplex. Angew. Chem. Int. Ed. 2018; 57:15395–15399. 10.1002/anie.201809328 PMC6563693 30222243

